# Factors influencing local signs at catheter insertion site regardless of catheter-related bloodstream infections

**DOI:** 10.1186/s13054-021-03490-z

**Published:** 2021-02-17

**Authors:** Niccolò Buetti, Stéphane Ruckly, Jean-Christophe Lucet, Olivier Mimoz, Bertrand Souweine, Jean-François Timsit

**Affiliations:** 1 INSERM, IAME, University of Paris, 75006 Paris, France; 2 AP-HP, Infection Control Unit, Bichat- Claude Bernard University Hospital, 46 rue Henri Huchard, 75877 Paris Cedex, France; 3 Medical and Infectious Diseases Intensive Care Unit, AP-HP, Bichat-Claude Bernard University Hospital, 46 rue Henri Huchard, 75877 Paris Cedex, France; 4 Services Des Urgences Adultes and SAMU 86, Centre Hospitalier Universitaire de Poitiers, Université de Poitiers, 86021 Poitiers, France; 5 Inserm U1070, Poitiers, France; 6 Medical ICU, Gabriel-Montpied University Hospital, Clermont-Ferrand, France; 7 Infection Control Program and WHO Collaborating Centre On Patient Safety, Hospitals and Faculty of Medicine, University of Geneva, Geneva, Switzerland

To the editor,

Little is known on the role of local signs at the catheter exit site [1–3]. Using a large cohort with high-quality data from four randomized-controlled trials we recently showed that local signs at insertion site (*i.e.*, a composite endpoint including redness, pain, purulent and non-purulent discharge) were significantly associated with catheter-related bloodstream infections (CRBSI) [4]. However, a question remains open: Which factors may influence local signs regardless of CRBSI? To our knowledge, no data in the recent literature are available.

We therefore re-analyzed our large cohort with 6976 patients and 14,590 short-term catheters, and we used as a primary endpoint “ ≥ 1 local sign.” We used multivariable logistic regression in order to identify variables associated with ≥ 1 local sign. Logistic models were stratified for the different centers included in the analysis.

Importantly, patients over 75 years (OR 0.82, 95% CI 0.72–0.94, *p* = 0.0044), with high SOFA score (OR 0.66, 95% CI 0.55–0.79, *p* < 0.001), immunosuppression (OR 0.72, 95% CI 0.59–0.88, *p* = 0.0014), catheter duration ≤ 7 days (OR 0.30, 95% CI 0.27–0.34, *p* < 0.001), and jugular (OR 0.62, 95% CI 0.49–0.80, *p* = 0.0001) or femoral (OR 0.76, 95% CI 0.64–0.90, *p* = 0.0012) sites significantly decreased the risk to develop local signs (Table 1) regardless of CRBSI. Clinicians should deserve particular attention to these specific populations of critically ill patients, who may decrease the risk of developing local signs. Among patients with CRBSI (*n* = 114), severely injured patients (*i.e.*, with high SOFA score or under vasoactive medications), immunosuppressed patients and femoral catheters had fewer local signs (data not shown).

**Table 1:** Risk factors of having ≥ 1 local sign (multivariable logistic regression)

	OR	95% CI	*p* value	
**CRBSI**	**4.242**	**2.811**	**6.402**	**< 0.0001**
Male sex	1.093	0.981	1.218	0.11
Age > 75 years*	**0.823**	**0.719**	**0.941**	**0.0044**
SOFA score*				
SOFA 12–14	0.777	0.665	0.908	0.0015
SOFA 9–11	0.852	0.742	0.977	0.022
SOFA > 14	**0.660**	**0.552**	**0.790**	** < 0.0001**
Immunosuppression	**0.719**	**0.587**	**0.881**	**0.0014**
Vasopressor at inclusion	1.043	0.916	1.187	0.52
Catheter days ≤ 7	**0.303**	**0.273**	**0.336**	** < 0.0001**
Catheter type, CVC (*versus* AC)	1.057	0.875	1.277	0.57
Experience of the operator < 50 procedures	0.945	0.842	1.062	0.34
*Insertion site*				
Jugular	**0.623**	**0.488**	**0.796**	**0.0001**
Subclavian	1.018	0.801	1.292	0.89
Femoral	**0.755**	**0.637**	**0.895**	**0.0012**
Vasopressor at insertion	0.961	0.853	1.083	0.52
Antibiotic at insertion	**1.271**	**1.138**	**1.420**	** < 0.0001**

Bold: statistically significant

*The log linearity was not respected for SOFA and age, and therefore, we created two qualitative variables.

CRBSI: catheter-related bloodstream infection. OR: odds ratio. CI: confidence interval. IQR: interquartile range. ICU: intensive care unit. SOFA: Sequential Organ Failure Assessment. CVC: central venous catheter. AC: arterial catheter.

In our previous analysis, we found that local signs observed within the first 7 catheter-days are predictive for intravascular catheter infections [4]: We are convinced that especially in this subgroup clinicians should be aware of the frequent absence of local signs in elderly, severe, immunosuppressed patients, and jugular/femoral catheters in the decision-making process.

Interestingly, pathological temperature (body temperature ≥ 38.5 °C or ≤ 36.5 °C), catheter type, and severity of illness in the presence of local signs did not help clinician in predicting intravascular catheter infections [4]. In light of all these considerations, we summarized in Table 2 practical clinical implications that may help ICU specialists when dealing with local signs and suspicion of intravascular catheter infections.

**Table 2:** Practical clinical implications

Factors that independently decreased local signs at insertion site:	Older age Severe ill patients Immunosuppression Catheter maintenance ≤ 7 days Jugular and femoral sites
Factors that decreased local signs in patients with CRBSI	Severe ill patients Immunosuppression Femoral site
Factors influencing the management of catheter*	Redness, non-purulent discharge, and purulent discharge are significantly associated with CRBSI Local signs are absent in almost 60% of CRBSI Local signs observed within the first 7 days are highly predictive for intravascular catheter infections Pathological temperature (body temperature ≥ 38.5 °C or ≤ 36.5 °C), catheter type, and severity of illness in the presence of local signs do not help clinician in predicting intravascular catheter infections

*see reference [4]

CRBSI: catheter-related bloodstream infection.

## Data Availability

The datasets used and/or analyzed during the current study are available from the corresponding author on reasonable request.

## Abbreviations

CRBSI: Catheter-related bloodstream infections
CVC: Central venous catheter
ICU: Intensive care unit
OR: Odds ratio
SOFA: Sequential organ failure assessment
